# Difficult Access and Poor Productivity: Mammography Screening in Brazil

**DOI:** 10.31557/APJCP.2019.20.6.1857

**Published:** 2019

**Authors:** Danielle Cristina Netto Rodrigues, Ruffo Freitas-Junior, Rosemar Macedo Sousa Rahal, Rosangela da Silveira Corrêa, João Emílio Peixoto, Noely Vicente Ribeiro, Nilson Clementino Ferreira, Leonardo Ribeiro Soares

**Affiliations:** 1 *Brazilian Network for Breast Research, Advanced Breast Diagnosis Center (CORA), School of Medicine, *; 3 *Institute of Social and Environmental Studies (IESA), *; 4 *School of Civil and Environmental Engineering, Federal University of Goiás, Goiânia, Goiás, *; 2 *Brazilian Network for Breast Research, Service for Quality Control in Ionizing Radiation, National Cancer Institute (INCA), Rio de Janeiro, RJ, Brazil. *

**Keywords:** Mammography, diagnostic screening programs, universal health insurance, national health programs

## Abstract

**Background:**

Factors that may hamper access to mammographic screening in any given region include socioeconomic limitations and the geographical distribution and quality of the mammography machines. This study evaluated access to breast cancer screening within the Brazilian National Health Service (SUS), the geographical distribution of mammography equipment and the number of mammograms performed in Brazil.

**Methods:**

This ecological study evaluated the availability of mammography machines within the SUS, those available for Brazil as a whole, its macro-regions, states and the Federal District in 2016. The number of mammography machines required for breast cancer screening was calculated and compared to the number of machines available. The expected number of mammograms was compared with the actual number performed. Machines were georeferenced based on their location and the municipal seat, according to healthcare region, with 60 km being defined as the maximum distance for an individual to travel for a mammogram.

**Results:**

In 2016, there were 4,628 mammography machines in Brazil. Of these, 4,492 were in use and 2,113 (47%) were available to the SUS. Considering the number of mammograms required as a function of the number clinically indicated, 2,068 machines would be required for breast cancer screening in Brazil. The network of machines available would be capable of producing 14,279,654 exams; however, only 4,073,079 exams were performed, representing 29% of the total capacity of production in the country in 2016. Regarding the maximum distance of 60 km to access a mammogram, only relatively small areas of Brazil were found not to meet this indicator.

**Conclusion:**

These results suggest that the difficulty of the Brazilian population in accessing breast cancer screening through the SUS is not associated with the number of machines available or with the geographical location of the equipment but rather with the insufficient number of mammograms performed.

## Introduction

Breast cancer is the most common form of cancer in women and the major cause of death from cancer in this group (Bray et al., 2018). Breast screening and early diagnosis can reduce mortality rates and the morbidity associated with treatment (Berry et al., 2005; Soares and Freitas-Junior, 2018). Nevertheless, to be considered effective, a population-based screening program must periodically encompass at least 70% of the target population (WHO, 2018).

Several factors may hamper access to mammography in a given geographical region, including socioeconomic inequalities and the geographical distribution and quality of the equipment (Lima-Costa and Matos, 2007; Vieira et al., 2017). In developing countries, the problem is compounded by the fact that human resources are limited, as are the consumables required for the equipment to function adequately, ultimately affecting productivity (Sopelete and Biscarde, 2013; Toledo et al., 2016; Vieira et al., 2017).

In Brazil, women’s healthcare programs and services began to be implemented following the introduction of policies aimed at democratizing and decentralizing healthcare in the country (Paim et al., 2011; Passman et al., 2011). The “Pact for Health” (Brasil, 2006) gave rise to a new phase of development within the Brazilian National Health Service (Sistema Único de Saúde, SUS), with a focus on regionalization, negotiation and agreement in the processes of political and territorial organization, leading to the formation of geographical healthcare regions (Vianna et al., 2010; Lima et al., 2012a; Lima et al., 2012b).

Currently, there is a regional planning director for each Brazilian state and for the Federal District, a strategy that divides the territory into health regions, promoting decentralization and improving the management of various public health sectors. In addition, it permits analyses more closely focused on planning and on the required interventions, including access to mammography (Brasil, 2002; Viacava et al., 2012).

Each healthcare region is expected to ensure that a sufficient number of mammography machines are available to meet the requirements of the target population, taking into consideration a maximum distance of 60 kilometers (Brasil, 2015; Amaral et al., 2017) between the equipment and the place of residence of this population. Nonetheless, no systematic evaluations of breast cancer screening coverage within the SUS or of the difficulties experienced in accessing mammography in Brazil have yet been performed. Therefore, the objective of this study was to evaluate access to mammography and the number of mammograms performed within the Brazilian public healthcare system, as well as the geographical distribution of mammography machines.

## Materials and Methods

This was an ecological study in which the unit of observation was the number of mammography machines available to the SUS and to the resident population of Brazil, its macro-regions, states and the Federal District in 2016.

Data on the number of mammography machines were collected from the National Register of Health Establishments (CNES/DATASUS) and refer to data available in October 2016 (Brazil, 2016a). Both analog and digital mammography machines were taken into consideration. Regarding the resident population and women in the specified age range, data on the projected population for 2016 were extracted from the Brazilian Institute of Geography and Statistics (IBGE) database (IBGE, 2013).

*Study area*

The areas analyzed in the present study were Brazil as a whole, its macro-regions, states and the Federal District. Brazil is a country of continental proportions, with a total area of 8,515,767,049 square kilometers (IBGE, 2016). For 2016, the Brazilian population was estimated at 206 million inhabitants (IBGE, 2013), distributed across five macro-regions: the north, northeast, southeast, south and Midwest of the country. These macro-regions are divided into 26 states and a Federal District, which, in turn, encompass 438 health regions (Brazil, 2002).

*Calculation of the number of mammography machines required*

The number of mammography machines required to meet the demands of the target population was calculated according to Ministry of Health Ordinance GM/MS No. 1631 issued on October 1, 2015, which takes into consideration the age group for which screening is indicated and the capacity of production of the equipment (Brazil, 2015). The expected number of mammograms per year was calculated according to equation 1 and the required number of machines was calculated using equation 2:

Equation 1

NM = D1 + OI + BCS +D2

where:

NM = necessary number of mammograms/year.

D1 = number of diagnostic mammograms to be performed (10% of the female population in the 40-49-year age group).

OI: other indications for performing a mammogram (10% of the female population in the 40-49-year age group).

BCS: breast cancer screening (50% of the female population in the 50-69-year age group).

D2: number of diagnostic mammograms to be performed (8.9% of the female population in the 50-69-year age group).

Equation 2

Nm = NM/6,758 

where:

Nm = necessary number of mammography machines/year.

NM = necessary number of mammograms/year.

6,758 = 80% of the capacity of production of exams/year per machine (Nelson et al., 2016).

*Evaluation of access*

Sixty kilometers was defined as the maximum distance an individual should have to travel to undergo mammography, respecting the territorial limits of the health regions (Brasil, 2015; Amaral et al., 2017). Based on this parameter, the areas in which the population has access to the equipment available within the SUS network were outlined to evaluate the geographical access of the target population to the exam.

The machines were georeferenced according to their location and the municipal seat. The geographical coordinates were obtained from the data linked to the IBGE code for the municipality and mapping was performed using the ArcGIS spatial analyst software, version 10.2.2.

*Performance of the exams*

The capacity of production of exams within the SUS network, calculated according to Equation 1, was compared with the number of mammograms performed in 2016. The number of mammograms performed was obtained from the DATASUS outpatient data system (SIA) using the variable “approved value” provided by the State Health Departments (Brasil, 2016b).

*Statistical analysis*

To evaluate the association between the distribution of the female population aged 50 to 69 years and the number of mammography machines in each health region, the two variables were normalized to values ranging from 0 to 100 in which the health region with the smallest female population of 50-69 years of age was awarded a value of 0 and the largest a value of 100. The same was performed for the number of mammography machines. Following normalization of these two variables, linear regression analysis was performed.

## Results

According to the CNES/DATASUS data, there were 4,628 mammography machines registered in Brazil in 2016. Of these, 4,492 were in use and 2,113 (47%) were available for use within the SUS network. Of those available to the SUS, 6% were in the north of the country, 28% in the northeast, 39% in the southeast, 18% in the south and 8% in the Midwest.

**Figure 1:**
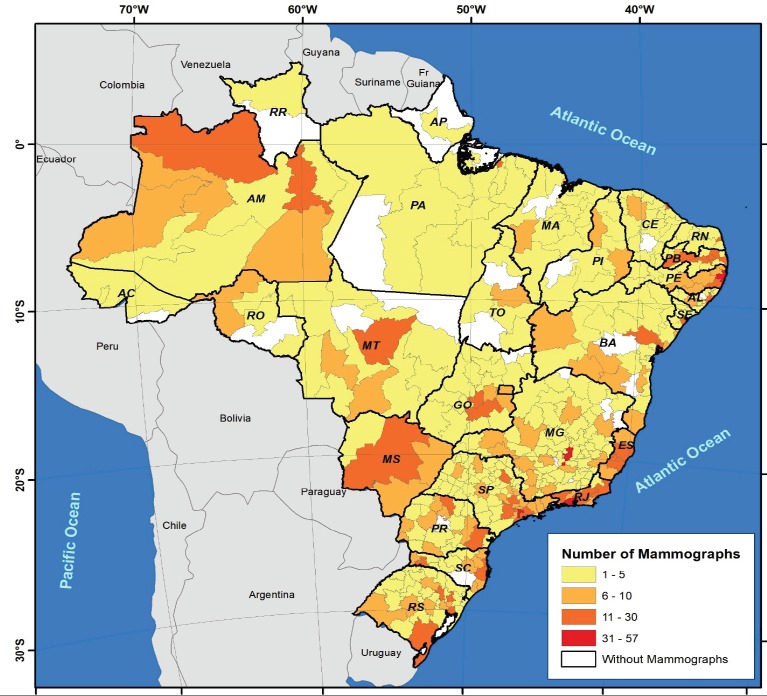
Distribution of Mammography Machines According to Health Regions in the States and in the Federal District: Brazil, 2016

In the individual states and the Federal District, the number of machines available ranged from 3 in Acre and Roraima to 391 in São Paulo. Mammography machines were available for use within the SUS in 405 (92.5%) of the 438 health regions in the country, with the number of machines ranging from 1 to 57 in each region in which there was equipment (Figure 1). 

Consolidation of the data from the health regions per states and the Federal District, macro-regions and the entire country showed that 2,068 machines would be required to meet the demands of the target population defined in the Ministry of Health’s Ordinance GM/MS 1631. Although 2,113 machines were available within the SUS network, reflecting a surplus of 45 machines, when the macro-regions were analyzed individually a deficit of 141 devices was found for the southeast of the country (Table 1).

A similar deficit was found when the states and the Federal District were analyzed individually, with an insufficient number of machines being found in nine states. In six of these states (Roraima, Acre, Pará, Maranhão, Rio de Janeiro and São Paulo), only 60-79% of the total number of machines required was actually available and in another three (Amapá, Ceará and Paraná) between 80 and 90% of the required number was actually available. Findings were similar in the Federal District where only 66% of the recommended number of machines was available.

**Figure 2:**
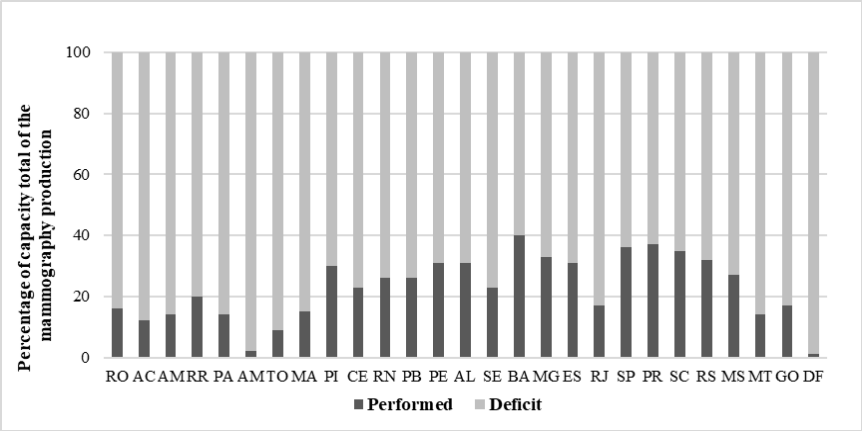
Comparison between the Number of Mammograms Performed and the Total Capacity of the Mammography Machines Available to the Brazilian National Health Service (SUS) per state and the Federal District: Brazil, 2016

Although the number of machines available to the SUS in Brazil is theoretically sufficient for 14,279,654 exams to be performed, only 4,073,079 mammograms were actually performed, representing 29% of the total production capacity in the country in 2016 (Table 1): 35% in the south of the country, 31% in the southeast, 30% in the northeast, 15% in the Midwest and 13% in the north. When stratified according to the individual states, production ranged from 2% of the total production capacity in Amapá to 40% in Bahia. In the Federal District, only 1% of the total production capacity of the machines available within the SUS network was achieved (Figure 2).

**Figure 3:**
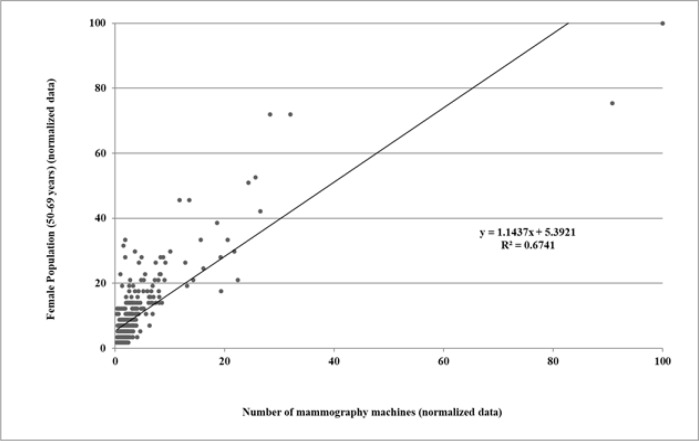
Linear Regression Analysis of the Female Population of 50 to 69 Years of Age and the Number of Mammography Machines According to Health Region: Brazil, 2016

As shown in Figure 3, the value obtained in the regression analysis (R2=0.6741) indicates that the association between the geographical distribution of the mammography equipment and the population living in the health regions is reasonably precise. The angular coefficient with a value close to 1 (1.1437) indicates that the accuracy of distribution is excellent, with the intercept of 5.3921 showing a weak tendency towards the population being slightly too big to be served by the number of machines available in the health regions.

**Figure 4:**
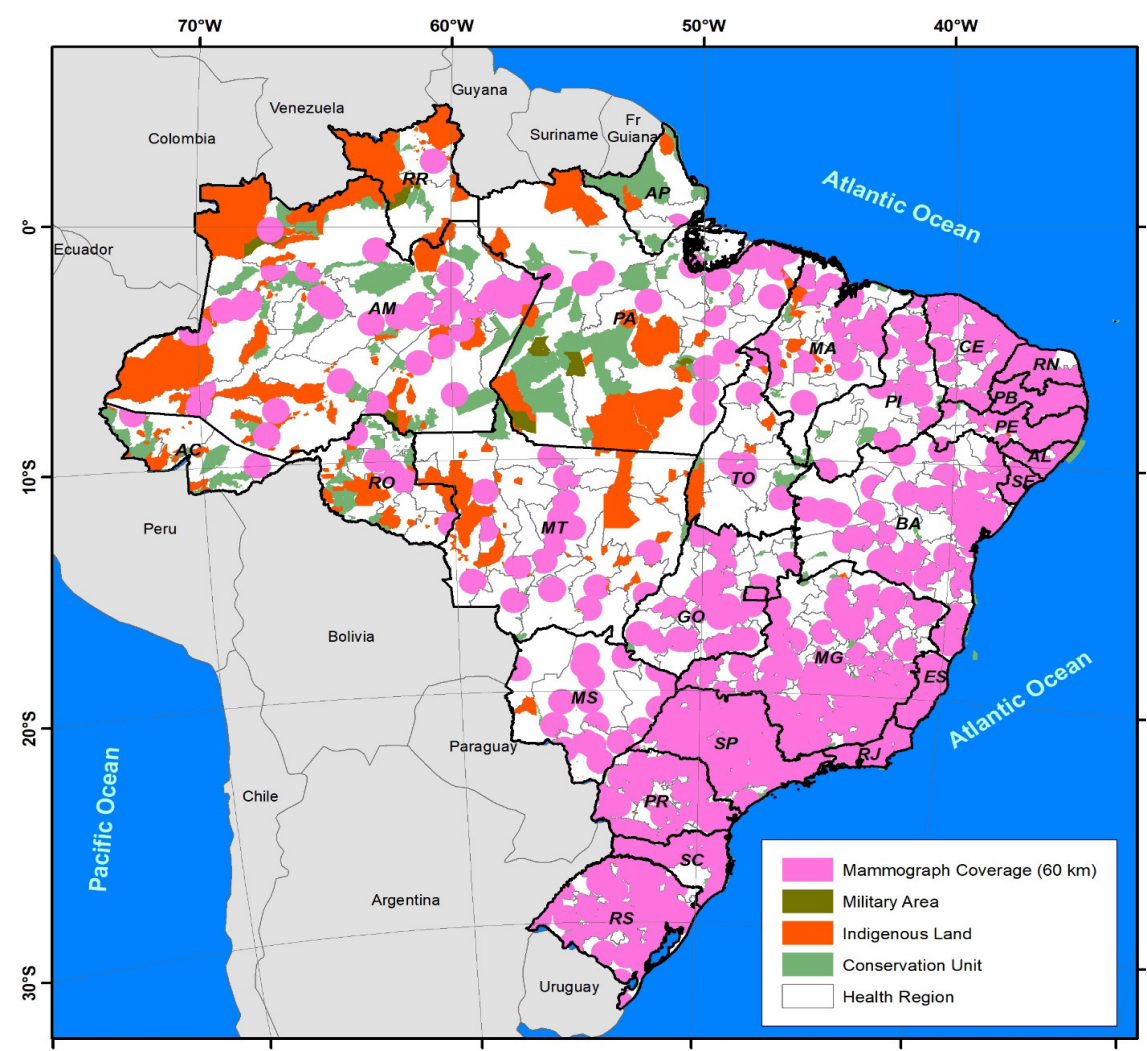
Spatial Coverage Provided by the Network of Mammography Machines Available to the Brazilian National Health Service (SUS), and Environmental Conservation Areas, Military Zones and Land Inhabited by Indigenous Peoples According to Health Regions: Brazil, 2016

Based on the parameter of 60 kilometers as being the maximum distance between an individual’s home and a machine, with the territorial limits of the health regions in which there were machines being respected, the spatial coverage of the network of mammography machines available for use within the SUS network in 2016 was found to be complete in the southern and southeastern macro-regions of the country and in several of the states in the northeast. On the other hand, coverage was incomplete in the north and Midwest (Figure 4).

**Figure 5:**
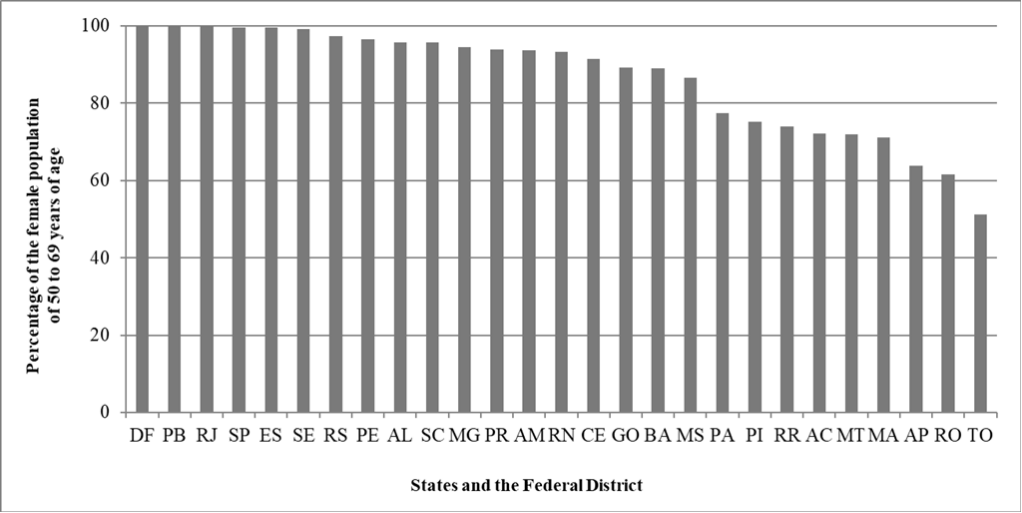
Percentage of the Female Population of 50 to 69 Years of Age Living within 60 Kilometers of a Mammography Machine Available to the Brazilian National Health Service (SUS) per State and the Federal District: Brazil, 2016

The machines available within the SUS network currently serve 81% of Brazilian municipalities, with 4,502 of the municipal seats being within the area of spatial coverage. Analyzing the size of the target population residing within 60 kilometers of a mammography machine, it was found that spatial coverage encompassed 94% of this population. Figure 5 shows the evaluation of the distance of 60 kilometers per state.

**Table 1:** The Number of Mammography Machines Needed and the Number Available in Brazil, Its Macro-Regions, States and the Federal District in 2016

State, Federal District / Macro-Region	Number of mammograms performed	Number of mammograms needed*	Number of mammography machines needed*	Number of mammography machines available
Rondônia	15,595	96,721	14	11
Acre	4,273	34,695	5	3
Amazonas	24,319	174,070	26	63
Roraima	4,297	21,711	3	3
Pará	55,245	392,253	58	37
Amapá	763	31,267	5	4
Tocantins	7,097	77,685	11	14
North	111,589	828,403	123	135
Maranhão	49,109	338,420	50	35
Piauí	59,555	195,780	29	34
Ceará	123,698	544,577	81	68
Rio Grande do Norte	56,771	217,492	32	33
Paraíba	65,210	253,377	37	118
Pernambuco	188,013	607,272	90	97
Alagoas	58,817	191,844	28	38
Sergipe	30,288	132,580	20	26
Bahia	375,925	931,243	138	147
Northeast	1,007,386	3,412,584	505	596
Minas Gerais	495,734	1,503,407	222	259
Espírito Santo	83,458	271,190	40	44
Rio de Janeiro	231,324	1,350,886	200	125
São Paulo	1,214,733	3,365,379	498	391
Southeast	2,025,249	6,490,862	960	819
Paraná	309,302	825,095	122	118
Santa Catarina	172,450	497,532	74	101
Rio Grande do Sul	295,686	920,400	136	169
South	777,438	2,243,027	332	388
Mato Grosso do Sul	46,948	173,192	26	33
Mato Grosso	27,398	193,519	29	47
Goiás	75,679	433,241	64	85
Distrito Federal	1,392	197,660	29	10
Midwest	151,417	997,612	148	175
Brazil	4,073,079	13,972,489	2,068	2,113

* Based on Ordinance GM/MS No. 1,631, of October 1, 2015. (Brasil, 2015).

## Discussion

In the present study, the difficulties involved in accessing breast cancer screening were stratified into various well-established quantitative parameters, permitting critical analysis of Brazil’s current situation. Mammography coverage within the SUS is presently around 25%, as reported in several population-based studies (Correa et al., 2011; Freitas-Junior et al., 2016). Consequently, monitoring mammography coverage may help in the elaboration of public policies aimed at consolidating breast cancer screening. 

The geographical factor and the need for an individual to travel a considerable distance to undergo screening limit access to breast cancer screening. In countries of continental dimensions such as Brazil, this limitation may hamper actions aimed at early breast cancer detection (Sopelete and Biscarde, 2013; Toledo et al., 2016). Nevertheless, in this study, the geospatial evaluation conducted by health region showed that 94% of the target population had access to screening. On the other hand, evaluation according to each state and the Federal District showed that spatial coverage failed to reach 70% of the target population only in the states of Tocantins, Rondônia and Amapá, all situated in the north of the country.

The use of the indicator of 60 kilometers’ distance to define geographical access should be viewed with caution, since an individual would have to travel this distance at least four times - there and back to undergo examination and there and back once again to obtain the results of the exam. Therefore, according to the government, having to travel 240 kilometers would be acceptable. However, in real life, this total distance could represent an important barrier preventing women from undergoing breast cancer screening, a situation further aggravated by the difficulties posed by educational, financial and organizational barriers.

The present study identified an unequal distribution of mammography machines across the country, with a surplus of equipment in 17 states and a deficit in 9 states and in the Federal District. These results could be explained by the methodology used in which the number of devices required was calculated based on the indicator number of devices required for a target population in accordance with the current parameters established by the Ministry of Health (Brasil, 2015). 

Therefore, the present findings suggest that the poor mammography coverage in Brazil is not related to a lack of equipment or to the geographical distribution of the equipment. Nevertheless, our findings are in agreement with those of previous studies showing that the number of mammograms performed is low (Viana et al., 2010; Tabar et al., 2011; Lima et al., 2012a; Sopelete and Biscarde, 2013; Tomazelli and Silva, 2017).

This low productivity is not in line with the capacity of the mammography machines available within the country. In 2016, there was capacity for 14,279,654 mammograms, while only 4,073,079 exams were performed. Therefore, only 29% of the target population was able to access mammography, while another 9,899,410 exams failed to be carried out despite the existence of the necessary equipment. The lack of human resources and, in many cases, a lack of the consumables required for breast screening to be adequately performed are additional factors that hamper productivity (Brasil, 1990; Lima-Costa & Matos, 2007; Toledo et al., 2016; Vieira et al., 2017).

Although the number of exams performed was small, according to government figures the Brazilian National Health Service paid around R$184 million for mammograms in 2016 (Rodrigues et al., 2013). In view of the investments made and the importance of mammography for the early detection of breast cancer, more vigorous public health policies are required to ensure access of the population to good quality mammograms with the lowest possible risk (Viacava et al., 2012).

Measures were adopted to minimize the limitations of this study, since there is a possibility that some of the mammography devices registered in the National Health Service database could have been counted twice (analog, stereotaxis, and/or digital). In addition, issues such as devices embargoed by an official organ or broken devices could have led to an overestimation of the number of machines available.

Finally, it has to be taken into consideration that the principles established by the SUS of universal and integrated healthcare depend on various determinants, including, principally, reducing social inequalities (Viana et al., 2010; Youlden et al., 2012). Therefore, considering all the investments in health and the advances made in relation to increasing offer within the network of services made available by the SUS, it is clear that the population’s access to these healthcare services, which is affected by the social inequalities determined by the idiosyncrasies of each region, continues to represent a challenge for public policies in the country (Viana et al., 2010; Youlden et al., 2012).

In conclusion, access remains difficult and the production of mammograms within the Brazilian public healthcare system is insufficient. The spatial coverage of the network of mammography machines could be considered adequate despite inequalities in the geographical distribution of these machines.

## Conflicts of Interest

The authors declare that they have no competing interests.

## References

[B2] Barros MB (2017). Social inequality in health: revisiting moments and trends in 50 years of publication of RSP. Rev Saude Publica.

[B3] Berry DA, Cronin KA, Plevritis SK (2005). Cancer Intervention and Surveillance Modeling Network (CISNET) Collaborators Effect of screening and adjuvant therapy on mortality from breast cancer. N Engl J Med.

[B4] Biller-Andorno N, Jüni P (2014). Abolishing mammography screening programs? A view from the Swiss Medical Board. N Engl J Med.

[B5] Bray F, Ferlay J, Soerjomataram I (2018). Global cancer statistics 2018: GLOBOCAN estimates of incidence and mortality worldwide for 36 cancers in 185 countries. CA Cancer J Clin.

[B6] Coldman A, Phillips N, Wilson C (2015). Pan-Canadian study of mammography screening and mortality from breast cancer. J Natl Cancer Inst.

[B15] Corrêa RS, Freitas-Junior R, Peixoto JE (2011). Estimated mammogram coverage in Goiás State, Brazil. Cad Saude Publica.

[B16] Corrêa RS, Freitas-Junior R, Peixoto JE (2012). Effectiveness of a quality control program in mammography for the Brazilian National Health System. Rev Saude Publica.

[B17] d’Avila Viana AL, Lima LD, Ferreira MP (2010). Structural conditions for regionalization in health care: typology of Regional Management Boards. Cien Saude Colet.

[B18] Freitas-Junior R, Rodrigues DCN, Corrêa RS (2016). Contribution of the Unified Health Care System to mammography screening in Brazil, 2013. Radiol Bras.

[B19] Gøtzsche PC, Jørgensen KJ (2013). Screening for breast cancer with mammography. Cochrane Database Syst Rev.

[B22] Jørgensen KJ, Gøtzsche PC, Kalager M, Zahl PH (2017). Breast cancer screening in Denmark: a cohort study of tumor size and overdiagnosis. Ann Intern Med.

[B23] Lima-Costa MF, Matos DL (2007). Prevalence and factors associated with mammograms in the 50-69-year age group: a study based on the Brazilian National Household Sample Survey (PNAD-2003). Cad Saude Publica.

[B24] Lima LD, Queiroz LFN, Machado CV, Viana AL (2012a). Decentralization and regionalization: dynamics and conditioning factors for the implementation of the Health Pact in Brazil. Cien Saude Colet.

[B25] Lima LD, Viana AL, Machado CV (2012b). Regionalization and access to healthcare in Brazilian states: historical and political-institutional conditioning factors. Cien Saude Colet.

[B26] Miller AB, To T, Baines CJ, Wall C (2002). The Canadian National Breast Screening Study – 1: breast cancer mortality after 11 to 16 years of follow-up A randomized screening trial of mammography in women aged 40 to 49 years. Ann Intern Med.

[B27] Morris E, Feig SA, Drexler M, Lehman C (2015). Implications of overdiagnosis: impact on screening mammography practices. Popul Health Manag.

[B28] Nelson HD, Fu R, Cantor A (2016). Effectiveness of breast cancer screening: systematic review and meta-analysis to update the 2009 US Preventive Services Task Force Recommendation. Ann Intern Med.

[B29] Paim J, Travassos C, Almeida C, Bahia L, Macinko J (2011). The Brazilian health system: history, advances, and challenges. Lancet.

[B30] Passman LJ, Farias AM, Tomazelli JG (2011). SISMAMA: implementation of an information system for breast cancer early detection programs in Brazil. Breast J.

[B31] Rodrigues DCN, Freitas-Junior R, Corrêa RS (2013). Performance of diagnostic centers in the classification of opportunistic screening mammograms from the Brazilian public health system (SUS). Radiol Bras.

[B32] Soares LR, Freitas-Junior R (2018). The impact of mammography screening on the surgical treatment of breast cancer. Breast J.

[B33] Sopelete MC, Biscarde DGS (2013). Acesso aos serviços de saúde na realidade brasileira: sugestões para superação de alguns desafios Access to health services in the Brazilian reality: suggestions for overcoming some challenges. Rev Enc Pesq Educ.

[B34] Tabár L, Vitak B, Chen TH (2011). Swedish two-county trial: impact of mammographic screening on breast cancer mortality during 3 decades. Radiology.

[B35] Thiede M, McIntyre D (2008). Information, communication and equitable access to health care: a conceptual note. Cad Saude Publica.

[B36] Toledo SR, Almeida NA, Souza MR, Minamisava R, Freitas-Junior R (2016). Care flow of breast cancer patients in the public health care network. Rev Eletr Enf.

[B37] Tomazelli JG, Silva GA (2017). Breast cancer screening in Brazil: an assessment of supply and use of Brazilian National Health System health care network for the period 2010-2012. Epidemiol Serv Saude.

[B38] Viacava F, Ugá MA, Porto S, Laguardia J, Moreira RS (2012). Evaluation of performance of health systems: a model for analysis. Cien Saude Colet.

[B39] Vieira RA, Formenton A, Bertolini SR (2017). Breast cancer screening in Brazil Barriers related to the health system. Rev Assoc Med Bras.

[B40] Youlden DR, Cramb SM, Dunn NA (2012). The descriptive epidemiology of female breast cancer: an international comparison of screening, incidence, survival and mortality. Cancer Epidemiol.

[B41] World Health Organization (2007). Early Detection. Cancer control: knowledge into action: WHO guide for effective programmes; module 3.

